# *lsaC* and Tandem *lsaE-lnuB* Resistance Genes in Invasive Group A *Streptococcus*

**DOI:** 10.3201/eid3203.251776

**Published:** 2026-03

**Authors:** Bernard Beall, Saundra Mathis, Zhongya Li, Joy Rivers, Anne-Kathryn Venero, Benjamin J. Metcalf, Lesley McGee, Sopio Chochua

**Affiliations:** Applied Science, Research & Technology, Inc., Atlanta, Georgia, USA (B. Beall, S. Mathis, Z. Li, A.-K. Venero); Centers for Disease Control and Prevention, Atlanta (J. Rivers, B.J. Metcalf, L. McGee, S. Chochua)

**Keywords:** Streptococci, bacteria, antimicrobial resistance, group A Streptococcus, antibiotic resistance, pleuromutilins, clindamycin, mobile elements, recent acquisitions of resistance, United States

## Abstract

Among >16,500 recently recovered invasive *Streptococcus pyogenes* isolates, we detected 9 independent acquisitions of *lsaC* or tandem *lsaE-lnuB* genes, which are known to confer resistance to pleuromutilins and clindamycin. Continued awareness of the evolving *S. pyogenes* antimicrobial resistosome is important for future infection treatment considerations.

Group A *Streptococcus* (GAS) commonly causes noninvasive infections affecting the skin and throat and invasive infections that can involve any tissue of the human body. Treatment of GAS infections is primarily with β-lactam antimicrobial drugs; macrolides and clindamycin are alternatives for patients allergic to β-lactam antimicrobial drugs (*1*). GAS co-resistance to macrolides and clindamycin has increased (*2*), which compromises macrolide usage for noninvasive infections and combined clindamycin with penicillin for severe disease (*1*). The 2 main streptococcal macrolide resistance mechanisms are 23S rRNA methylation by *erm* gene–encoded methylases, which confers resistance to macrolides, lincosamides (including clindamycin), and streptogramin B antimicrobials, and macrolide efflux by *mef-*encoded and *msrD*-encoded proteins (*3*). The *lnu* genes confer lincosamide resistance, whereas *lsa* genes confer resistance to lincosamides, streptogramin A drugs, and pleuromutilins. The pleuromutilin lefamulin is approved in the United States for systemic treatment of community-acquired bacterial pneumonia in adults (*4*) and has potent antibacterial activity against β-hemolytic streptococci (*5*). Although *lsa* and *lnu* genes are documented in group B *Streptococcus* (*6*,*7*) only 1 GAS isolate carrying tandem *lsaE-lnuB* determinants has been reported (*8*).

We identified 11 invasive GAS (iGAS) blood isolates positive for *lsa* or *lnu* genes, 7 *lsaC* and 4 *lsaE/lnuB*, from >16,500 iGAS isolates recovered during 2015–2023 and 335 isolates screened before 2015 through Active Bacterial Core surveillance (ABCs). We detected the positive isolates on 1 of 9 distinct mobile elements (Figures 1, 2; Appendix Table, Figures 1–9). Each element was found within 1 of 7 different iGAS strains (7 *emm* type/sequence type [ST] combinations; for example, *emm89.0/*ST101). Two strains were represented by indistinguishable (360807 and 360907) or nearly identical (20156709 and 20175626) isolate genome sequences available obtained under BioProject PRJNA395240 (Appendix Table).

**Figure 1:**
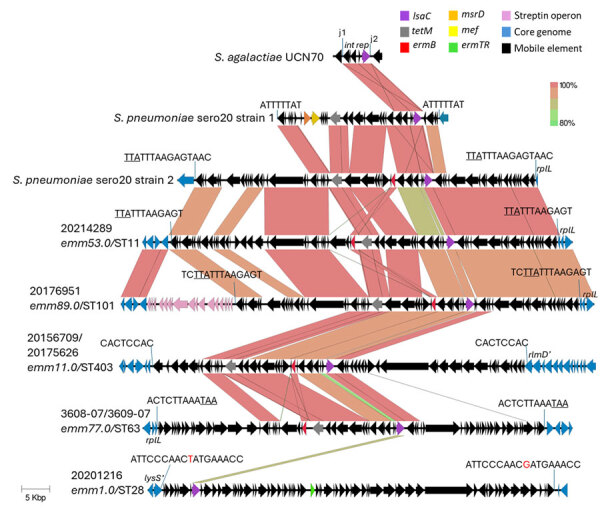
Alignments of 5 different group A *Streptococcus* *lsaC*-carrying accessory elements from study of repeated acquisitions of *lsaC* and tandem *lsaE-lnuB* resistance genes. Alignments include a partial element from *S. agalactiae* strain UCN70 (*6*) and 2 complete elements recently described in pneumococci (*9*). The j1 and j2 (junctions 1 and 2) sequences depict 24–25 bp sequences that demarcate a 5,258–5,816 bp mobilizable *lsaC*-carrying cassette that is highly conserved between all of the strains shown except for iGAS strain 20201216 (Appendix Figure 1, panel B). The 8–18 bp target sequence repeat flanking each complete element shown is perfect except in strain 20201216 (nonconserved base in red font). Underlined text indicates the stop codon of the *rplL* gene in 4 strains (including *S. pneumoniae* strain 2). The insertion within strain 20156709/20175626 targeted an 8-bp internal sequences within the *rlmD* gene, resulting in a truncated allele, *rmlD’*; the insertion within strain 20201216 targeted an 18-bp internal sequence within the *lysS* gene, resulting another truncated allele, *lysS’*. Scale bar indicates 5,000 base pairs. ST, sequence type.

**Figure 2:**
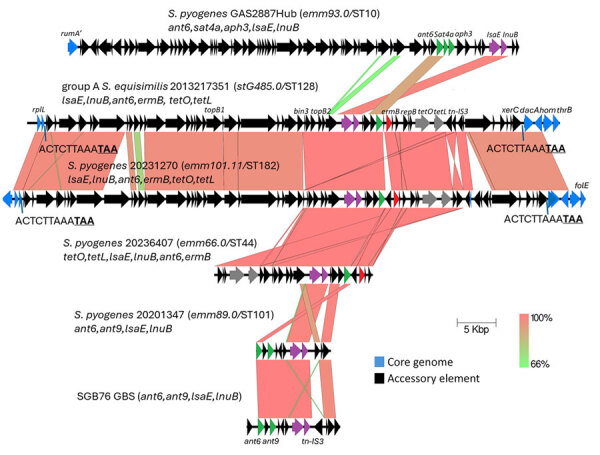
Alignment of complete *lsaE-*carrying elements from study of repeated acquisitions of *lsaC* and tandem *lsaE-lnuB* resistance genes by group A *Streptococcus.* Alignments shown are from group A ABCs strains 2013217351 and 20231270 with partial elements from strains 20236407 and 20201347. Also included are complete elements from GAS2887Hub (*8*) and GBS strain SGB76 (GenBank accession no. KF772204). Antimicrobial resistance genes include 3 aminoglycoside 6-adenyltransferase genes (*ant6, aph3,* and *ant9*), and the streptothricin acetyltransferase gene *aph3.* Prokka annotations include *topB* (DNA topoisomerase genes), *bin3* (DNA invertase gene), *repB* (DNA replication gene), *tn-IS3* (IS3 family transposase gene), and *xerC* (tyrosine recombinase gene). Underlined bold text indicates the stop codon of the *rplL* gene in 2 strains. Scale bar indicates 5,000 base pairs. ABCs, Active Bacterial Core surveillance; sero, serotype; ST, sequence type.

We found 5 large (61,501–78,917 bp) accessory elements carrying *lsaC* in combination with *ermB* and *tetM* in 6 isolates from 5 strains and with *ermTR* in isolate 20201216 (Figure 1). All 6 elements were flanked by short genomic target repeats, indicative of genomic insertion through precisely targeted transposition (*7*). Three of the 5 elements were inserted at the *rplL* 3′ end, 1 within the *rlmD* gene, and 1 within the *lysS* gene.

Four iGAS strains, including 1 *S. equisimilis* isolate (*10*), carried an identical *lnuB* allele and conserved *lsaE* alleles sharing 98%–100% sequence identity on 4 distinct accessory elements (Appendix Figure 2, panel B). As with 3 *lsaC-*carrying elements (Figure 1), 2 of the 4 elements carrying *lsaE-lnuB* mapped at the *rplL* 3′ end and were also apparently inserted through precise transposition events. For 2 strains, we were unable to map element genomic insertion sites because of incomplete assembly.

The 4 deduced 492 residue LsaE proteins shared 52.2%–53.7% sequence identity with the 5 deduced 494 residue LsaC protein sequences. Other than resistance determinants, few genes were conserved between the 4 mobile elements carrying *lsaE-lnuB* from this study with the prophage described from *S. pyogenes* strain Gas2887Hub (*8*) also carrying those genes (Figure 2). The iGAS *S. equisimilis* strain 2013217351 and *S. pyogenes* 20231270 carried closely related transposons, each inserted at the 12-mer *rplL* 3′ terminus conserved between the 2 species.

The 5 *lsaC-*carrying elements represented 4 phylogenetically distinct *lsaC* alleles (Appendix Figure 1) with 90.4%–99.8% sequence identity to the *S. agalactiae* UCN70 *lsaC* allele (*6*). The 4 elements carrying *lsaC*, *ermB*, and *tetM* each contained a small (5,258–5,816 bp) conserved *lsaC* self-mobilizing element inserted within a consensus Tn916 *ori*T site sequence (Appendix Figure 1, panel B) described in *S. agalactiae* (*7*) and recently described in 2 distinct pneumococcal elements (*9*). There was wide sequence divergence between the 4 small *ori*T-targeting iGAS *lsaC* mobile elements, despite identical 24–25 bp sequences flanking their insertion sites. For the *lsaC-*containing element in strain 20156709/20175626, genomic insertion and phylogenetic data were consistent with the sequential genomic insertion of a Tn916 family element before a more recent second precise insertion of the 5546 bp *lsaC–*carrying element into its *ori*T site (Appendix Figure 2, panels A, B). For 2 other *lsaC-*carrying elements, phylogenetic data suggested recent introduction of the complete composite element, consisting of a Tn916-related element carrying an integrated small *lsaC* element (Appendix Figures 3–4).

Ten of the 11 study isolates were resistant to both erythromycin and clindamycin (Appendix Table); that resistance is associated with the presence of *ermB* and *lsaC* (6 isolates), *ermTR* and *lsaC* (1 isolate), or *ermB*, *lsaE*, and *lnuB* (3 isolates). One strain, 20201347 (*lsaE+*, *lnuB+*), was erythromycin susceptible but clindamycin-resistant, indicating *lsaE-* and *lnuB-*conferred clindamycin resistance. That finding in strain 20201347 was consistent with masking of *lsaE-* and *lnuB-*conferred clindamycin resistance in the other 10 isolates because of *erm* gene–encoded methylase activity. The 4 isolates carrying *lsaE-lnuB* had high MICs for the pleuromutilin lefamulin (MIC >2 µg/mL), whereas the 7 *lsaC-*positive isolates had low MICs for lefamulin (MICs <0.25 µg/mL) (Appendix Table). We conclude that expansion of *lsaE-*positive iGAS lineages could compromise future potential use of lefamulin, and *lsaC*- or *lsaE-lnuB–*positive strains could further undermine the use of clindamycin for treating β-hemolytic streptococcal infections.

Appendix Additional information about acquisition of *lsaC* and tandem *lsaE-lnuB* resistance genes in invasive group A *Streptococcus.*
