# Supplementary material for: lsaC and Tandem lsaE-lnuB Resistance Genes in Invasive Group A Streptococcus
Source: Emerg Infect Dis. 2026 Mar;32(3):465–9. doi: 10.3201/eid3203.251776 (PMC13016022; doi:10.3201/eid3203.251776)
Supplement: Appendix — Additional information about acquisition of lsaC and tandem lsaE-lnuB resistance genes in invasive group A Streptococcus. [file 25-1776-Techapp-s1.pdf]

Article DOI: https://doi.org/10.3201/eid3203.251776

# *lsaC* and Tandem *lsaE-lnuB* Resistance Genes in Invasive Group A Streptococci

## Appendix

## Supplementary Methods

### ABCs Data

Invasive group A streptococcal (iGAS) disease cases are defined through isolation of group A streptococci or detection of GAS DNA from a normally sterile site, or from a wound in an individual with necrotizing fasciitis or streptococcal toxic shock syndrome in an ABCs area resident (*1*). The surveillance sites included entire states or selected counties in 10 states, representing ≈30–35 million individuals during 2015–2023. Case medical charts were reviewed to attain demographic information. This activity was reviewed by the CDC Institutional Review Board, deemed not research, and was conducted consistent with applicable federal law and CDC policy (see e.g., 45 C.F.R. part 46.102(l) (*2*), 21 C.F.R. part 56; 42 U.S.C. §241(d); 5 U.S.C. §552a; 44 U.S.C. §3501 et seq.).

### ABCs Isolate Characterization

Isolates recovered during 2015–2023, as well were assigned *emm* subtypes, multilocus sequence types (STs), and resistance features employing short-read whole-genome sequencing as previously described (*2*) with genome sequences deposited in the National Center for Biotechnology Information Sequence Read Archive under BioProject number PRJNA395240. The 11 genome sequence accessions are listed in Appendix Table. Resistance determinant queries employed in house sequence queries that also included ResFinder and ARG-ANNOT sequence databases (*2–4*). Antimicrobial susceptibility testing was employed as described for verification of resistance phenotypes predicted for clindamycin, erythromycin, tetracycline (*2*,*5*), and the pleuromutilin lefamulin (*6*).

### Genomic Analyses

Prokka version 1.14.5 (*7*) was used to obtain annotated accessory elements (provided in supplement text files S1- S9), and sequences were then aligned to generate figures using EasyFig version 2.2.3 (*8*). Prokka-annotated DNA sequences for the 9 mobile elements described in this paper are available from the corresponding author upon request. Core genomic maximum parsimony genomic trees and pairwise single nucleotide polymorphism (SNP) matrices were generated from short-read

bacterial genome sequences employing kSNP4 (*9*) with a kmer size of 19. Resultant core.tre files generated from kSNP4 were used to generate core genomic pair-wise SNP matrices and phylogenetic diagrams employing Mega7 (*10*). Mafft (*11*) was used to align *lsaC* alleles before phylogenetic reconstruction employing FastTree (*12*) and Mega7.

**References**


1. ABCs Bactfacts Interactive Data Dashboard. Active bacterial core surveillance reports for 1997–2021. [cited 2024 Oct 13]. https://www. cdc.gov/abcs/bact-facts/data-dashboard.html
2. Chochua S, Metcalf BJ, Li Z, Rivers J, Mathis S, Jackson D, et al. Population and whole genome sequence based characterization of invasive group A streptococci recovered in the United States during 2015. MBio. 2017;8:e01422-17. PubMed https://doi.org/10.1128/mBio.01422-17
3. Gupta SK, Padmanabhan BR, Diene SM, Lopez-Rojas R, Kempf M, Landraud L, et al. ARG-ANNOT, a new bioinformatic tool to discover antibiotic resistance genes in bacterial genomes. Antimicrob Agents Chemother. 2014;58:212–20. PubMed https://doi.org/10.1128/AAC.01310-13
4. Zankari E, Hasman H, Cosentino S, Vestergaard M, Rasmussen S, Lund O, et al. Identification of acquired antimicrobial resistance genes. J Antimicrob Chemother. 2012;67:2640–4. PubMed https://doi.org/10.1093/jac/dks261
5. Clinical and Laboratory Standards Institute. Performance standards for antimicrobial susceptibility testing. 34th edition (M100). Wayne (PA): The Institute; 2024.
6. Paukner S, Riedl R. Pleuromutilins: potent drugs for resistant bugs—mode of action and resistance. Cold Spring Harb Perspect Med. 2017;7:a027110. PubMed https://doi.org/10.1101/cshperspect.a027110
7. Seemann T. Prokka: rapid prokaryotic genome annotation. Bioinformatics. 2014;30:2068–9. PubMed https://doi.org/10.1093/bioinformatics/btu153
8. Sullivan MJ, Petty NK, Beatson SA. Easyfig: a genome comparison visualizer. Bioinformatics. 2011;27:1009–10. PubMed https://doi.org/10.1093/bioinformatics/btr039
9. Hall BG, Nisbet J. Building phylogenetic trees from genome sequences with kSNP4. Mol Biol Evol. 2023;40:msad235. PubMed https://doi.org/10.1093/molbev/msad235
10. Kumar S, Stecher G, Tamura K. MEGA7: molecular evolutionary genetics analysis version 7.0 for bigger datasets. Mol Biol Evol. 2016;33:1870–4. PubMed https://doi.org/10.1093/molbev/msw054
11. Katoh K, Misawa K, Kuma K, Miyata T. MAFFT: a novel method for rapid multiple sequence alignment based on fast Fourier transform. Nucleic Acids Res. 2002;30:3059–66. PubMed https://doi.org/10.1093/nar/gkf436

12. Price MN, Dehal PS, Arkin AP. FastTree: computing large minimum evolution trees with profiles instead of a distance matrix. Mol Biol Evol. 2009;26:1641–50. PubMed https://doi.org/10.1093/molbev/msp077

13. Wescombe PA, Tagg JR. Purification and characterization of streptin, a type A1 lantibiotic produced by *Streptococcus pyogenes.* Appl Environ Microbiol. 2003;69:2737–47. PubMed https://doi.org/10.1128/AEM.69.5.2737-2747.2003

14. Brenciani A, Tiberi E, Bacciaglia A, Petrelli D, Varaldo PE, Giovanetti E. Two distinct genetic elements are responsible for *erm*(TR)-mediated erythromycin resistance in tetracycline-susceptible and tetracycline-resistant strains of *Streptococcus pyogenes.* Antimicrob Agents Chemother. 2011;55:2106–12. PubMed https://doi.org/10.1128/AAC.01378-10

Appendix Table. Features of 9 group A streptococcal strains (11 isolates) carrying *lsaC* or tandem *lsaE-lnuB* determinants*

| | | | | | | MIC, µg/mL (R, I, S, or U)¶ | | | | |
|---|---|---|---|---|---|---|---|---|---|---|
| Strain† | Year | *emm*/ST | *lsaC* or tandem *lsaE-lnuB* genes together with linked resistance genes on accessory element | Genomic insertion target repeat of element (relation of target to listed gene)‡ | Other resistance genes§ | Lef | Ery | Cli | Tet | Biosample accession number under BioProject PRJNA395240 (annotated element sequences available from author upon request) |
| 360807/ 360907 | 2006 | 77.0/63 | *lsaC,ermB,tetM* | *rplL* (12mer inclusive of stop codon) | *tetO* | ND ≤0.25(U)/ | >2 (R) >2 (R) | >1 (R) >1 (R) | >8 (R)/ >8 (R) | SAMN26504556 SAMN26504557 |
| 2013217351 | 2013 | stG485.0/128slv# (single locus variant of ST128) | *lsaE, lnuB, ermB, tetO,tetL,ant6* | *rplL* (12mer inclusive of stop codon) | No | >2 (U) | >2 (R) | >1 (R) | >8 (R) | SAMN27749685 |
| 20156709/ 20175626 | 2015/ 2017 | 11.0/403 | *lsaC,ermB,tetM* | *rlmD* internal 8mer | *aph3, sat4A* | ≤0.25 (U)/ ≤0.25 (U) | >2 (R) >2 (R) | >1 (R)/ >1 (R) | >8 (R)/ >8 (R) | SAMN07153834/ SAMN08692959 |
| 20176951 | 2017 | 89.0/101 | *lsaC,ermB,tetM* | *rplL* (14mer inclusive of stop codon) | no | ≤0.25 (U) | >2 (R) | >1 (R) | >8 (R) | SAMN08693232 |
| 20201216 | 2019 | 1.0/28 | *lsaC,ermTR* | *lysS* (internal 18mer, 1 mismatch) | no | ≤0.25 (U) | >2 | >1 (R) | ≤1 (S) | SAMN27862492 |
| 20201347 | 2019 | 89.0/101 | *lsaE,lnuB,ant6**,ant9*** | Undetermined due to incomplete assembly | *tetM, aph3, sat4A* | >2 (U) | ≤0.12 (S) | >1 (R) | >8 (R) | SAMN27862508 |
| 20214289 | 2021 | 53.0/11 | *lsaC,ermB,tetM* | *rplL* (12mer inclusive of stop codon) | no | ≤0.25 (U) | >2 (R) | >1 (R) | >8 (R) | SAMN27997403 |
| 20231270 | 2022 | 101.11/182 | *lsaE,lnuB,ermB,ant6,tetO,tetL* (S9) | *rplL* (12mer inclusive of stop codon) | no | >2 (U) | >2 (R) | >1 (R) | >8 (R) | SAMN41841278 |
| 20236407 | 2023 | 66/ST44slv | *lsaE,lnuB,ermB,ant6,tetO,tetL* (S10) | Undetermined due to incomplete assembly | no | >2 (U) | >2 (R) | >1 (R) | >8 (R) | SAMN45232838 |

*cli, clindamycin; ery, erythromycin; I, intermediate resistance; lef, lefamulin; ND, not done; R, full resistance; S, susceptible; U, undefined by Clinical and Laboratory Standards Institute (CLSI) for β-hemolytic streptococci; tet, tetracycline.

†Strains assigned with identifiers from independent iGASd case genome(s). Two strains were identified with two case isolate identifiers. Isolates 360807 and 360907 were genetically indistinguishable (0 single nucleotide polymorphisms (SNPs) and were recovered from two different adults (ages 51yr and 76yr) in the same county and on the same day. Isolates 20156709 and 20165626, although recovered during 2015 and 2017, respectively, differed by only 3 SNPs and shared an identical mobile element carrying *lsaC, ermB,* and *tetM.* These two isolates were recovered from two different adults (37yr and 73yr) residing within the same county.

‡Gene descriptions: *rplL*, ribosomal protein gene ; *rlmD*, RNA methyltransferase; *lysS*, lysine-tRNA ligase.

§*aph3* gene encodes aminoglycoside phosphorylase, *sat4a* encodes streptothricin N-acetyltransferase

¶Susceptibility.

#PubMLST/*S. dysgalactiae*

***ant6* and *ant9* are predicted to encode aminoglycoside nucleotidyltransferases

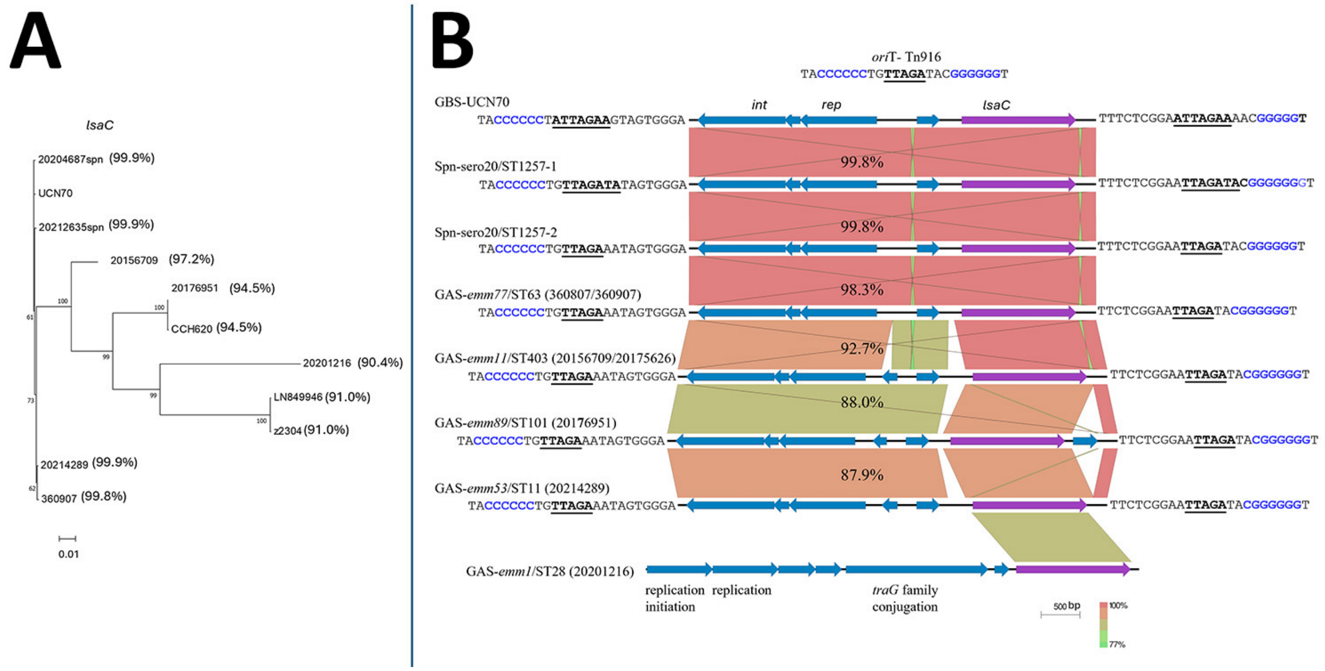


**Appendix Figure 1.** Phylogeny of *lsaC* alleles from different strains and alignment of small *lsaC* elements. A) Phylogenetic tree of *lsaC* alleles from GBS strains UCN70 and CCH620 (References 6 and 7 in main text), pneumococcal strains 20204687 and 20212635 (reference 10 in main text), and the 5 GAS *lsaC* alleles from this study. Percent sequence identities with reference allele from GBS-UCN70 are provided. *lsaC* cassettes integrated into different Tn916 family transposon *ori*T sites. B) These small elements from within larger composite elements shown in Figure 1 are enlarged here. Tandem repeats identical or similar to the underlined *ori*T region target are underlined and runs of Cs and Gs are indicated in purple. Percent identities of each GAS element to the GBS-UCN70 reference element are provided except for the divergent region from strain 20201216 (bottom). The flanking 24–25 bp sequences from the 4 GAS strains share complete identity. Levels of sequence identity are color coded according to bottom scale. Putative gene functions deduced from Prokka annotation and NCBI Blast searches are listed below strain 20201216 open reading frames.

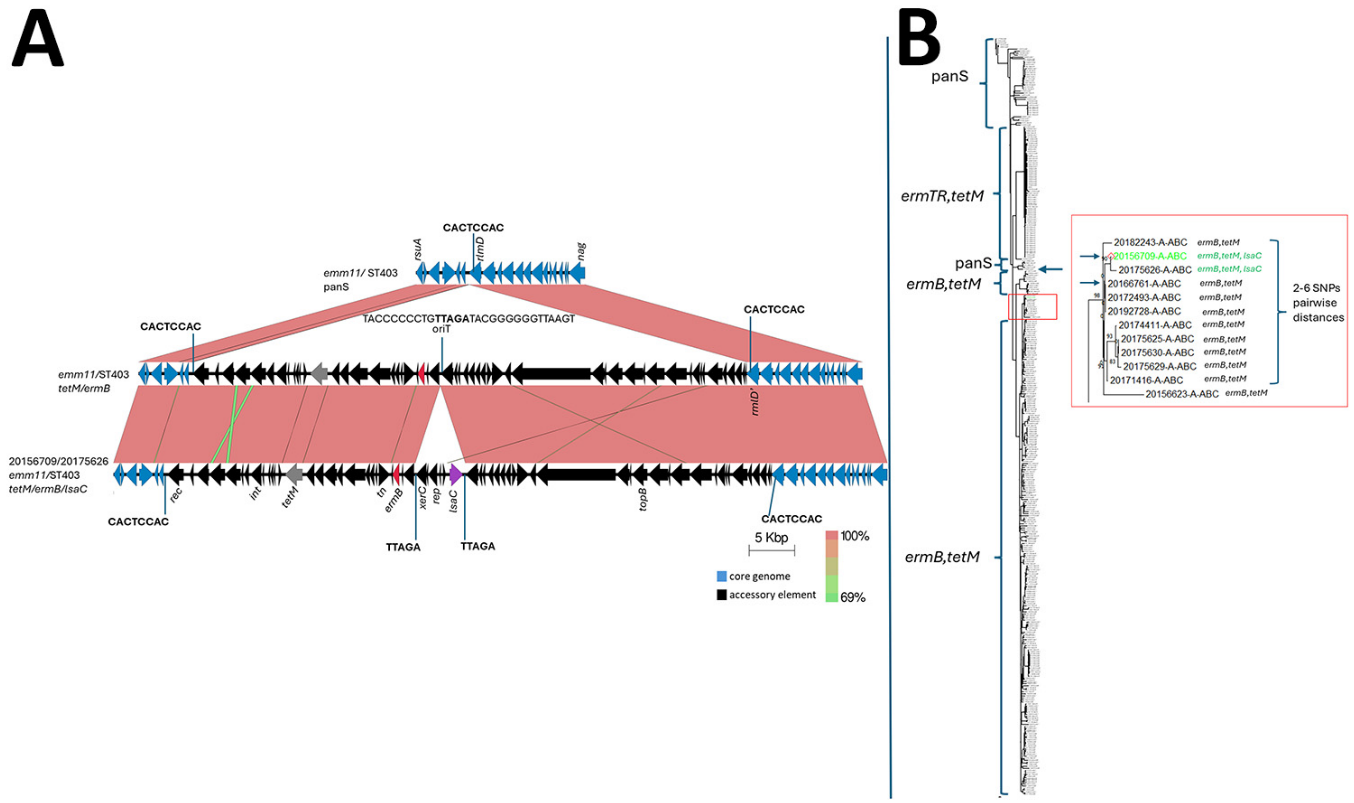


**Appendix Figure 2.** Evidence of insertion of a large Tn916 family mobile element carrying *ermB* and *tetM* followed by the second insertion of a smaller *lsaC*-carrying mobile element *lnu* in study of repeated acquisitions of *lsaC* and tandem *lsaE-lnuB* resistance genes by invasive group A streptococci. A) The top construct represents the first insertion event, depicted by the middle construct, where the 3′ internal *rlmD* 8mer target is repeated at the opposite end of the Tn916-related transposon. The second insertion event (bottom construct) targeted the Tn916 oriT sequence, with the 5 base insertion site repeated at each end of the 5546 bp *lsaC* element. Prokka-derived gene annotations have been added for selected core genomic and accessory factor open reading frame sequences. B) The parsimony core genome phylogeny tree was generated from 378 available *emm11.0/*ST403 ABCs isolates recovered during 2015–2019. There were 1,849 variable positions in the dataset. The left arrow corresponds to the strain depicted on top construct of Figure 1. Within the magnified red-outlined rectangle, strain 20156709/20175626 is depicted by top right arrow, with the bottom right arrow depicting the closest related ancestor *emm11/*ST403 (*tetM, ermB*), the theoretical recipient of the *lsaC* element.

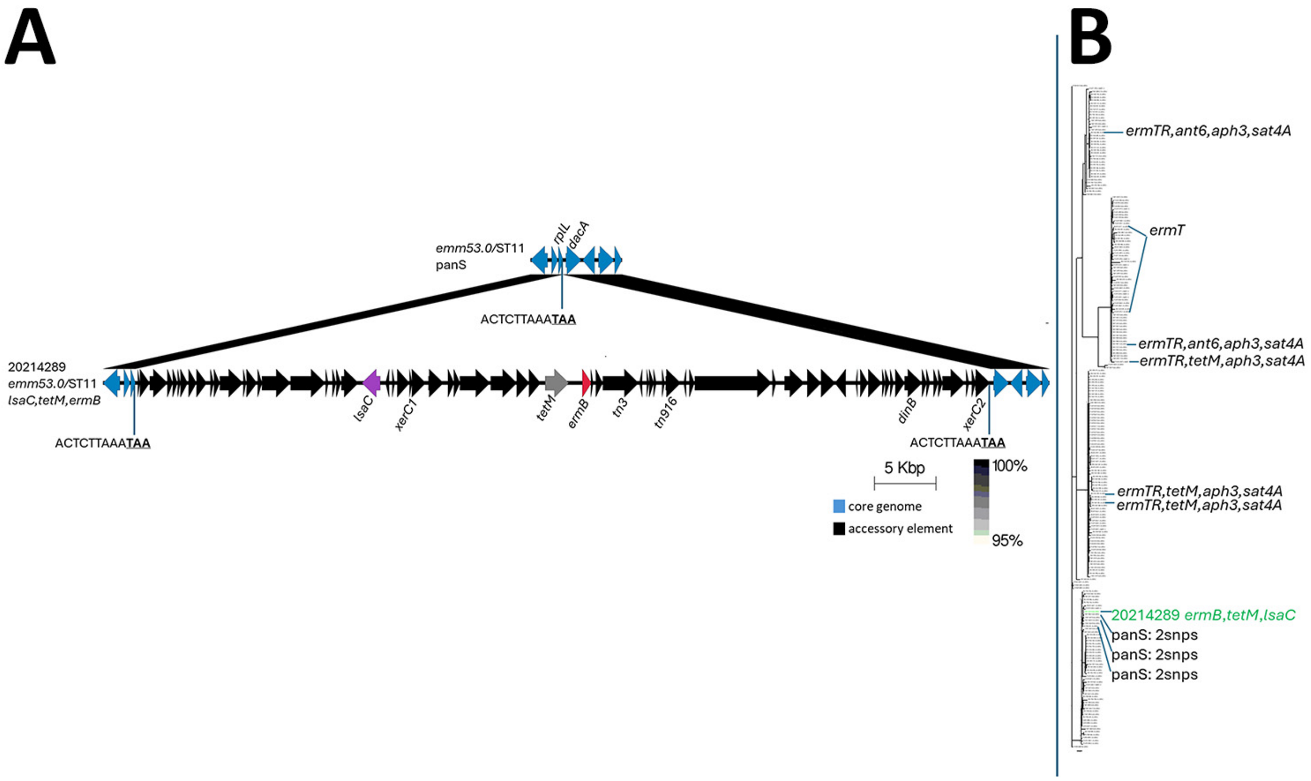


**Appendix Figure 3.** *lsaC*-containing element from *emm53.0/*ST11 strain 20214289. A) The top line shows the *rplL* insertion site with its 3′ repeated target consisting of the *rplL* 4 terminal codons (stop codon is underlined) repeated at both ends of the composite element (bottom line). Since disruption of the oriT site as shown in Appendix Figure 1 would presumably have prevented transposition, it is possible that the intermediate *ermB* and *tetM* -positive nearest ancestor representative of the first transposition event (as shown for strain 20156709/20175626 in Appendix Figure 2, panel A middle construct and panel B phylogeny) was not collected through ABCs. Alternatively, the composite element shown in strain 20214289 was introduced through an intra-species double-crossover homologous recombination event from a GAS strain already carrying the composite element. See Appendix Figure 1, panel B for more detail depicting the *lsaC* region. B) Strain 20214289 possibly received the composite 69,438 bp *ermB/tetM/lsaC* element in a single event since its core genome differed by only 2 SNPs from its nearest *emm53/*ST11 ancestors, which were pan-susceptible. The parsimony tree was generated from 226 available *emm53.0/*ST11 ABCs isolates There were 226 variable positions in the dataset. Isolates were pan-susceptible where not indicated.

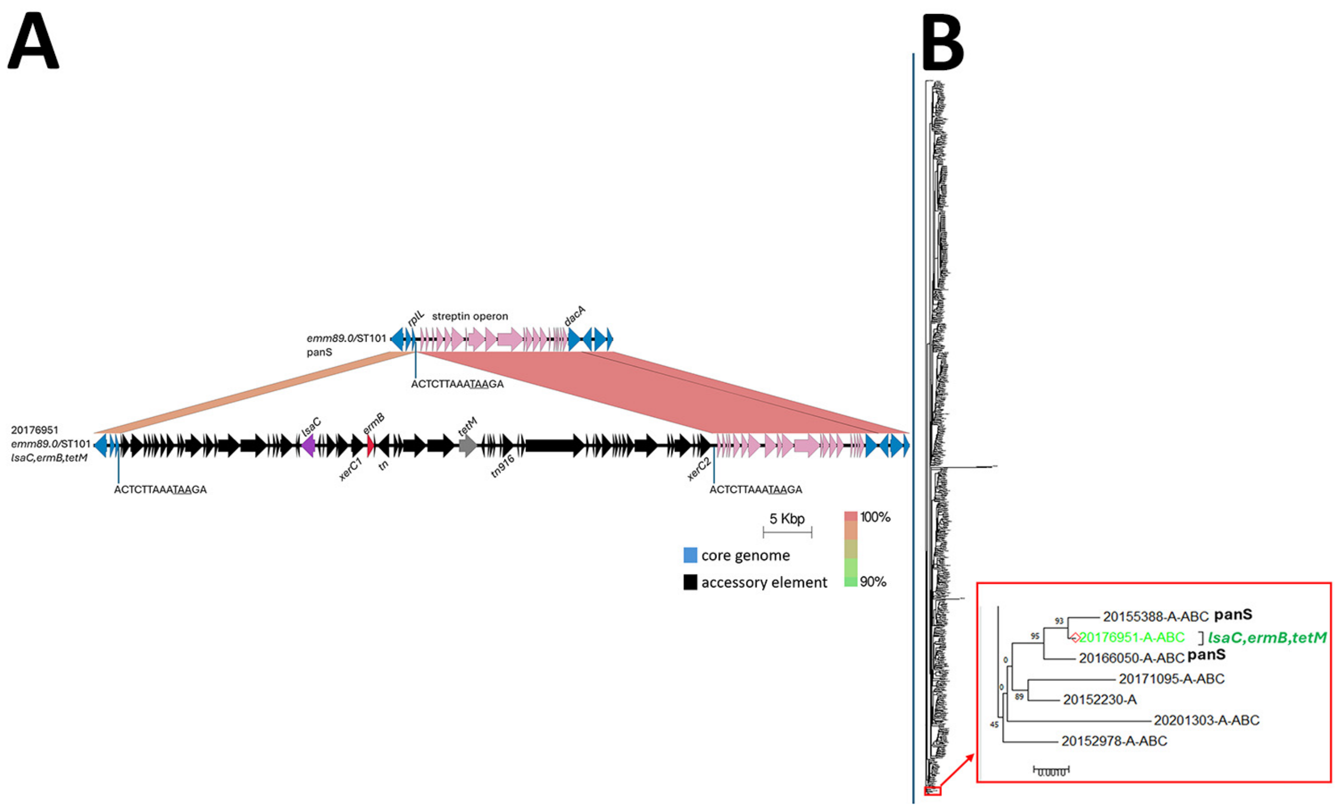


**Appendix Figure 4.** *lsaC*-positive strain 20176951. A) The 14 base target encompassing the *rplL* 4 terminal codons is shown in an ancestral related pan-susceptible strain (top construct) and is repeated at both ends of the composite element (bottom construct) upstream of the streptin biosynthetic operon which is variably present in *S. pyogenes* (*13*). See appendix Figure 1 panel B for more detail depicting the *lsaC* region. B) The parsimony tree was generated from 839 available *emm89.0/*ST101 ABCs isolates. Other than the *lsaC*+ isolate 20176951 recovered in Colorado, these isolates were recovered during 2015–2019 from ABCs areas other than Colorado. Isolates were pan-susceptible where not indicated. Preliminary analysis not shown revealed that the core genomes from 109 ABCs isolates recovered in Colorado differed by more than 20 SNPs from strain 20176051, while the two depicted pan-susceptible isolates recovered in Oregon (20155388 and 20166050) differed from strain 20176951 by only 8–10 SNPs.

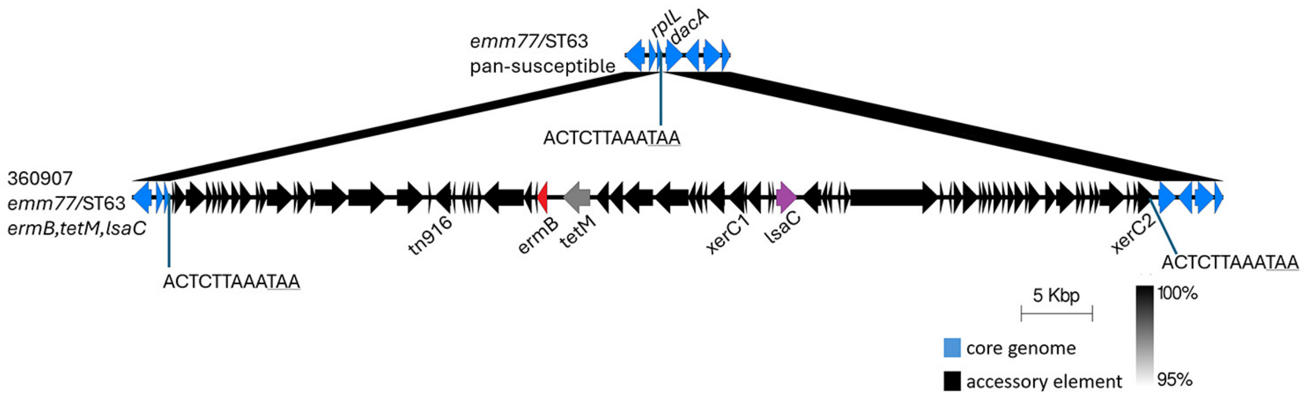


**Appendix Figure 5.** Element carrying *lsaC, ermB,* and *tetM* genes situated within the *emm77.0/*ST63 *rplL* 3′ end from isolate 360907 (Appendix Table). The *rplL* stop codon is underlined and constitutes the end 3 bases of the repeated targeted 12 base insertion site. A Tn916-related transposase gene (*tn916*) and two tyrosine recombinase family genes (*xerC1* and *xerC2*) are indicated. See Appendix Figure 1 panel B for more detail depicting the *lsaC* region.

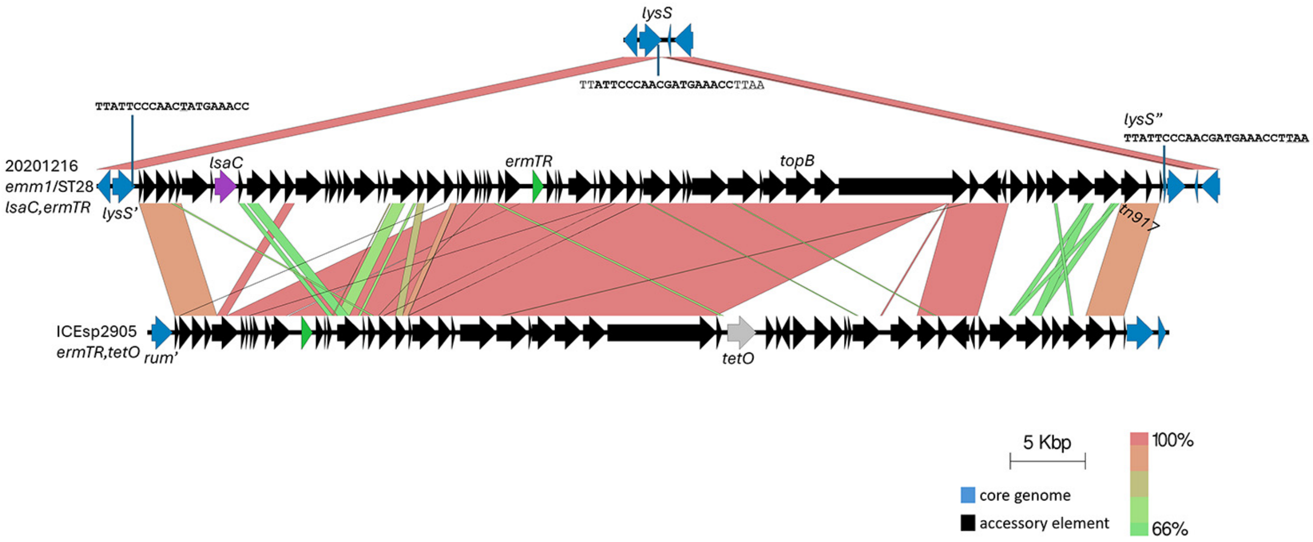


**Appendix Figure 6.** Insertion of element carrying *lsaC* and *ermTR* into the *lysS* 3′ end (*lysS* stop codon highlighted). The insertion in strain 20201216 is predicted to have fused 11 codons to the wild type *lysS* terminal coding sequence (not shown). A single base mismatch (silent) within the target site (bold) is indicated within the insertionally mutated *lysS* allele sequence (indicated as *lysS'*). Topoisomerase and *Tn916* transposase family genes annotated in Prokka are indicated. ICEsp2905 from *S. pyogenes* (*14*) carrying *ermTR* as does strain 20201216, and in addition *tetO*, was the closest sequence match to the element found in 20201216 and is depicted below. See appendix Figure 1 panel B for more detail that shows the divergence of the *lsaC* region compared to the other *lsaC*- carrying strains in this study.

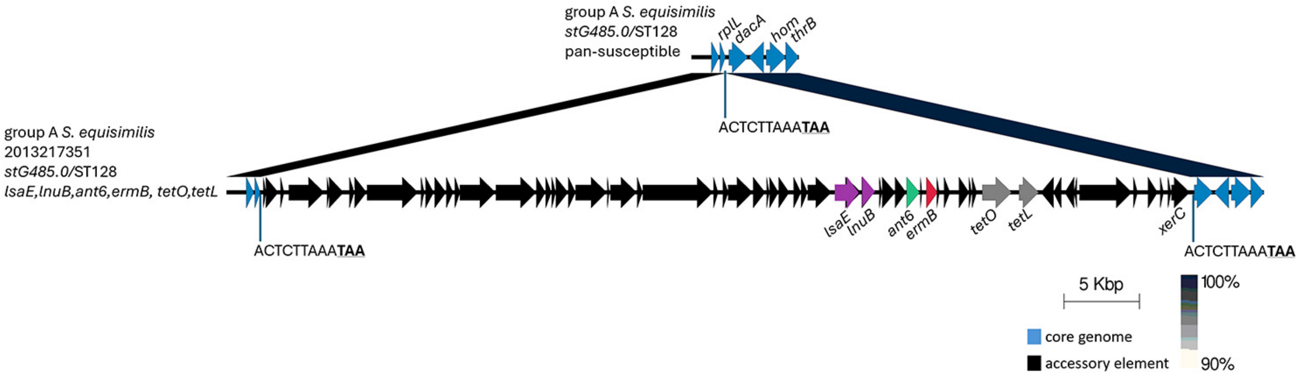


**Appendix Figure 7.** Alignment of year 2007 ABCs iGAS *S. equisimilis* strain 2013217351 *lsaE/lnuB* element insert region with same genomic region from a pan-susceptible iGAS *S. equisimilis stG485.0/*ST128 strain. The 4 terminal *rplL* codons (conserved between *S. pyogenes, S. equisimilis,* and *S. pneumoniae* (Figure 1) are repeated at the ends of the element.

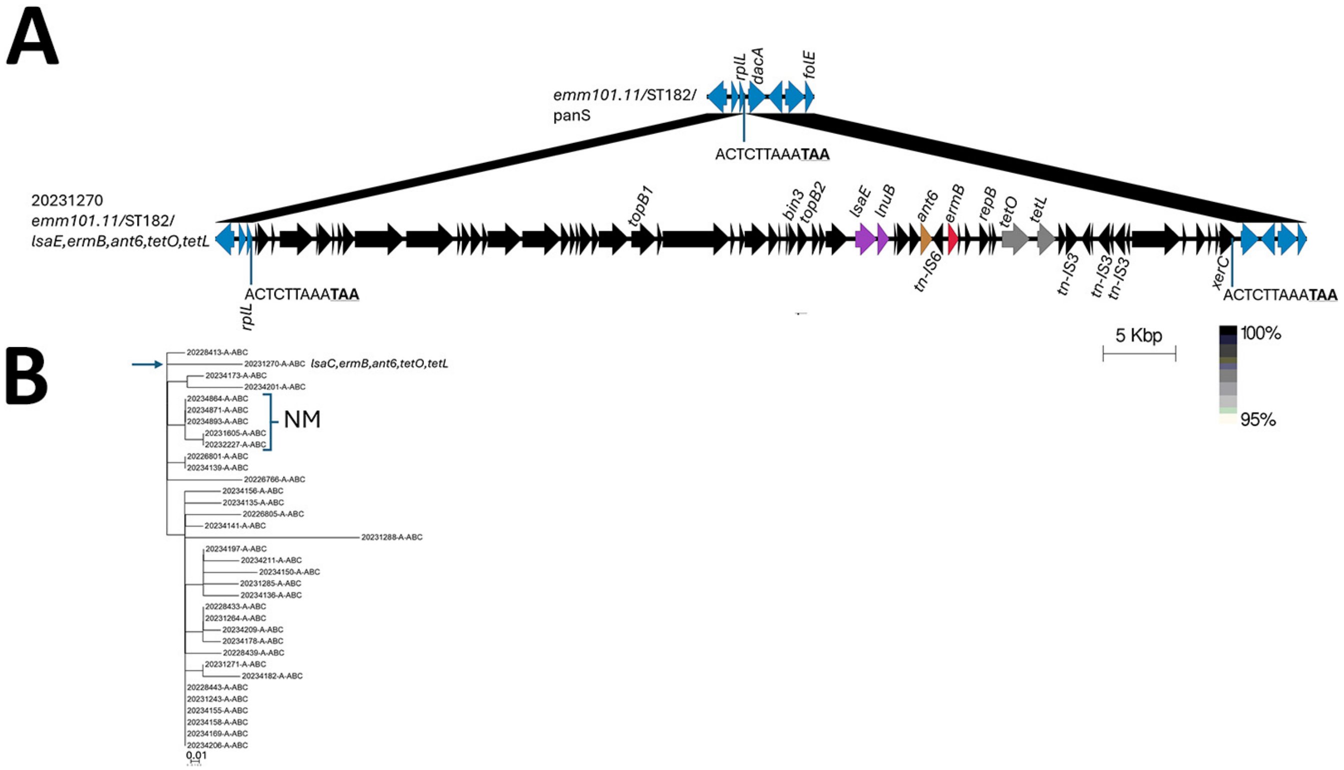


**Appendix Figure 8.** Evidence suggesting recent acquisition of the resistance element carrying *lsaE, lnuB, ermB, tetM, and tetL* found in ABCs *S. pyogenes* strain 20231270. A) Alignment of year 2022 ABCs *S. pyogenes* strain 20231270 *lsaC* element insert region with same genomic region from another *emm101.11/*ST182 isolate recovered during 2022. The repeated 4 codon repeat from the *rplL* terminus is shown. B) Phylogenetic tree of 35 available *emm101.11/* ST182 ABCs isolates recovered during 2022. All were recovered from Oregon with the exception of the indicated 5 isolate subcluster indicated from New Mexico (NM). The arrow indicates the position of *lsaE*-positive strain 20231270 which differs from the other 34 isolates by 4–14 SNPs in pairwise comparisons. There were only 53 variable positions in the 35 core genome dataset, also indicative of a close genomic cluster.

All were pan-susceptible with the exception of strain 20231270. Oregon ABCs strain 20231270 shared the new subtype *emm101.11* first noted in ABCs during 2022 with 34 other case isolates that were all recovered during 2022, however strain 20231270 was the only antibiotic-resistant isolate of this cluster, carrying the *lsaE, lnuB, ermB, ant6, tetO, and tetL* genes as shown. This observation, combined with the close relatedness of strain 20231270 to the other *emm101.11* isolates (4–14 core SNP differences), suggests recent acquisition of this resistance element.

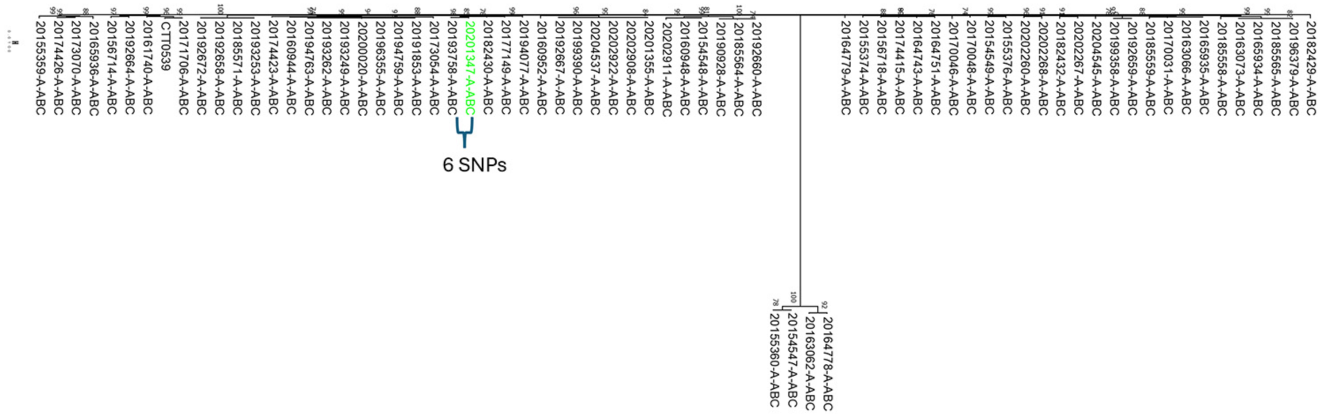


**Appendix Figure 9.** Core genome generated phylogenetic tree of 72 *emm89.0* ABCs isolates recovered in Connecticut during 2015–2019, including high lefamulin MIC (*lsaE*+) strain 20201347 (indicated in green) recovered during 2019 and differing from its closest neighbor recovered during 2018 by 6 SNPs. There were 1883 variable positions in the dataset.
